# Octa­butyl­bis{(*E*)-2-[4-(2-hydroxy­benzyl­ideneamino)phen­yl]acetato}di-μ_2_-methoxo-di-μ_3_-oxido-tetra­tin(IV)

**DOI:** 10.1107/S1600536809038938

**Published:** 2009-10-03

**Authors:** Wenkuan Li, Handong Yin, Liyuan Wen, Daqi Wang, Weidong Fan

**Affiliations:** a College of Chemistry and Chemical Engineering, Liaocheng University, Shandong 252059, People’s Republic of China.

## Abstract

The title compound, [Sn_4_(C_4_H_9_)_8_(C_15_H_12_NO_3_)_2_(CH_3_O)_2_O_2_], is a centrosymmetric dimer and displays a ladder type structural motif. There are four Sn^IV^ centres which can be divided into two sorts, *viz*. two endocyclic and two exocyclic. The endo- and exocyclic Sn^IV^ centres are linked by bidentate deprotonated methanol and μ_3_-O atoms. Each exocyclic Sn^IV^ centre is also coordinated by a monodentate 2-[4-(2-hydroxy­benzyl­idene­amino)phen­yl]acetate ligand. Parts of the butyl groups were found to be disordered over two sets of sites.

## Related literature

For related structures, see: Berceanc *et al.* (2002 ▶); Garcia-Zarracino & Hopfl (2005 ▶); Wu *et al.* (2009 ▶).
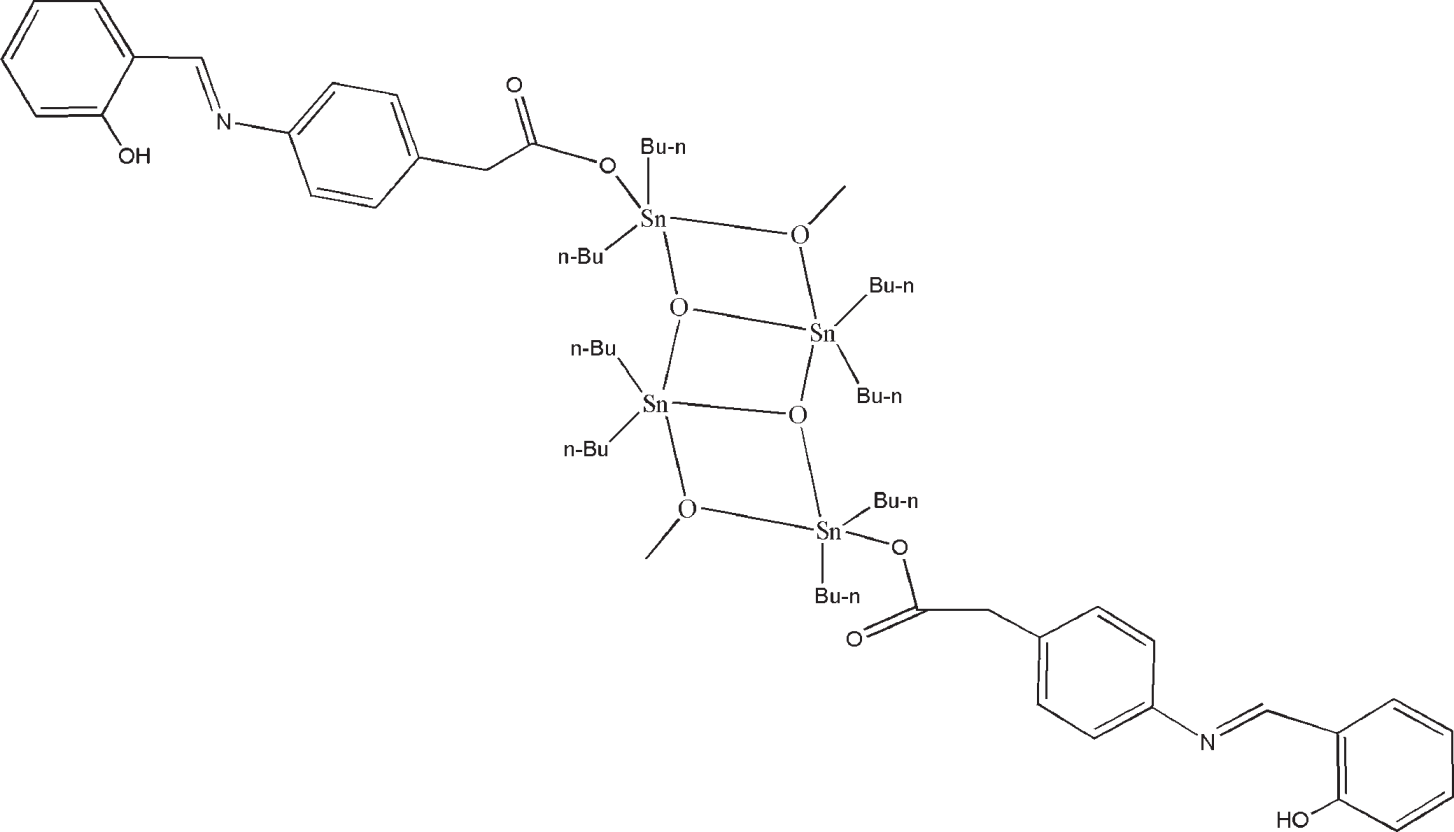


## Experimental

### 

#### Crystal data

[Sn_4_(C_4_H_9_)_8_(C_15_H_12_NO_3_)_2_(CH_3_O)_2_O_2_]
*M* *_r_* = 1534.32
Triclinic, 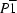

*a* = 10.6059 (14) Å
*b* = 12.2128 (17) Å
*c* = 14.522 (2) Å
α = 104.921 (2)°
β = 104.271 (2)°
γ = 91.991 (1)°
*V* = 1751.8 (4) Å^3^
*Z* = 1
Mo *K*α radiation
μ = 1.46 mm^−1^
*T* = 298 K
0.40 × 0.39 × 0.19 mm

#### Data collection

Siemens SMART CCD area-detector diffractometer
Absorption correction: multi-scan (*SADABS*; Sheldrick, 1996 ▶) *T*_min_ = 0.593, *T*_max_ = 0.769
9161 measured reflections
6067 independent reflections
4114 reflections with *I* > 2σ(*I*)
*R*_int_ = 0.019

#### Refinement

*R*[*F* ^2^ > 2σ(*F* ^2^)] = 0.035
*wR*(*F* ^2^) = 0.140
*S* = 1.04
6067 reflections
453 parameters
H-atom parameters constrained
Δρ_max_ = 1.31 e Å^−3^
Δρ_min_ = −0.56 e Å^−3^

### 

Data collection: *SMART* (Siemens, 1996 ▶); cell refinement: *SAINT* (Siemens, 1996 ▶); data reduction: *SAINT*; program(s) used to solve structure: *SHELXS97* (Sheldrick, 2008 ▶); program(s) used to refine structure: *SHELXL97* (Sheldrick, 2008 ▶); molecular graphics: *SHELXTL* (Sheldrick, 2008 ▶); software used to prepare material for publication: *SHELXTL*.

## Supplementary Material

Crystal structure: contains datablocks I, global. DOI: 10.1107/S1600536809038938/hg2567sup1.cif

Structure factors: contains datablocks I. DOI: 10.1107/S1600536809038938/hg2567Isup2.hkl

Additional supplementary materials:  crystallographic information; 3D view; checkCIF report

## Figures and Tables

**Fig. 1.**
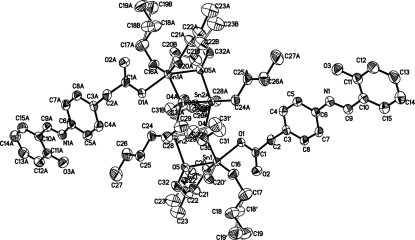
The molecular structure of (I), showing 50% probability displacement ellipsoids. H atoms have been omitted for clarity. [Symmetry code: -x + 1,-y + 1,-z]

## supplementary crystallographic information

### Comment

Recently, a large number of organotin compounds with different structural features have been reported in the literature (Berceanc *et al.*, 2002; Garcia-Zarracino & Hopfl  *et al.*, 2005). In continuation of our study of organotin compounds, we present here the synthesis and crystal structure of the title compound (I).

The title compound (Fig. 1) is a centrosymmetric dimer, with three linearly fused four-membered Sn—O—Sn—O rings, resulting in a ladder type structural motif. There are two tri-coordinate O atoms in (I), and each of them links two endocyclic tin atoms and one exocyclic tin atom (Sn(1)—O(4) = 2.022 (4) Å; Sn(2)—O(4) = 2.051 (4) Å; Sn(1)—O(4 A) = 2.127 (4) Å)(Table. 1). The additional links between the *endo*- and exocyclic Sn are provided by bidentate deprotonated methanol. Each exocyclic Sn atom is also coordinated by monodentate carboxylato ligand. Part of butyl groups were found to be disordered over two sites. The crystal structure of a similiar compound has been reported recently (Wu *et al.*, 2009).

### Experimental

The reaction was carried out under a nitrogen atmosphere. (*E*)-2-(4-(benzylideneamino)phenyl)acetic acid (1 mmol) and sodium ethoxide (1.2 mmol) were added to a stirred solution of benzene (30 ml) in a Schlenk flask and stirred for 0.5 h. Dibutyltin chloride (2 mmol) was then added to the reactor. After stirring for 6 h at 323 K, the yellow paste was obtained and then filtered. Yellow crystals suitable for X-ray analysis were obtained by slow evaporation of dichloromethane/methanol (1:1 V/V) solution over a period of six days (yield 86%. m.p.458 K). Anal. Calcd (%) for C_64_H_98_N_2_O_10_Sn_4_ (Mr = 1530.20): C, 50.23; H, 6.46; N, 1.83; O, 10.46; Sn, 31.03. Found (%): C, 50.19; H, 6.44; N, 1.85; O, 10.47; Sn, 31.05.

### Refinement

The atoms C18, C19, C20, C21, C22, C23, C29, C30 and C31 were found to be disordered over two sites, and the ratio of the occupancy factors refined to 0.70 (8):0.30 (8), 0.70 (8):0.30 (8), 0.66 (3):0.34 (3), 0.66 (3):0.34 (3), 0.66 (3):0.34 (3), 0.66 (3):0.34 (3), 0.66 (3):0.34 (3), 0.66 (3):0.34 (3) and 0.66 (3):0.34 (3) for atoms C18:*c*18', C19:*c*19', C20:*c*20', C21:*C*21', C22:*C*22', C23:*C*23', C29:C29', C30:*C*30' and C31:*C*31', respectively. H atoms were positioned geometrically, with C—H = 0.93, 0.96, 0.97 and 0.82 Å for aromatic, methyl, methylene and hydroxyl H atoms, respectively, and constrained to ride on their parent atoms, with *U*_iso_(H) = 1.2 *U*_eq_(C) or 1.5 *U*_eq_(C, O) for the methyl and hydroxyl groups

### Crystal data

**Table:**

[Sn_4_(C_4_H_9_)_8_(C_15_H_12_NO_3_)_2_(CH_3_O)_2_O_2_]	*Z* = 1
*M**_r_* = 1534.32	*F*(000) = 780
Triclinic, *P*1	*D*_x_ = 1.451 Mg m^−^^3^
Hall symbol: -P 1	Mo *K*α radiation, λ = 0.71073 Å
*a* = 10.6059 (14) Å	Cell parameters from 3867 reflections
*b* = 12.2128 (17) Å	θ = 2.2–25.7°
*c* = 14.522 (2) Å	µ = 1.46 mm^−^^1^
α = 104.921 (2)°	*T* = 298 K
β = 104.271 (2)°	Block, colorless
γ = 91.991 (1)°	0.40 × 0.39 × 0.19 mm
*V* = 1751.8 (4) Å^3^	

### Data collection

**Table:**

Siemens SMART CCD area-detector diffractometer	6067 independent reflections
Radiation source: fine-focus sealed tube	4114 reflections with *I* > 2σ(*I*)
graphite	*R*_int_ = 0.019
φ and ω scans	θ_max_ = 25.0°, θ_min_ = 1.7°
Absorption correction: multi-scan (*SADABS*; Sheldrick, 1996)	*h* = −9→12
*T*_min_ = 0.593, *T*_max_ = 0.769	*k* = −14→13
9161 measured reflections	*l* = −17→17

### Refinement

**Table:**

Refinement on *F*^2^	Primary atom site location: structure-invariant direct methods
Least-squares matrix: full	Secondary atom site location: difference Fourier map
*R*[*F*^2^ > 2σ(*F*^2^)] = 0.035	Hydrogen site location: inferred from neighbouring sites
*wR*(*F*^2^) = 0.140	H-atom parameters constrained
*S* = 1.04	*w* = 1/[σ^2^(*F*_o_^2^) + (0.0706*P*)^2^ + 2.6157*P*] where *P* = (*F*_o_^2^ + 2*F*_c_^2^)/3
6067 reflections	(Δ/σ)_max_ = 0.001
453 parameters	Δρ_max_ = 1.31 e Å^−^^3^
0 restraints	Δρ_min_ = −0.56 e Å^−^^3^

### Fractional atomic coordinates and isotropic or equivalent isotropic displacement parameters (Å^2^)

**Table:**

	*x*	*y*	*z*	*U*_iso_*/*U*_eq_	Occ. (<1)
Sn1	0.65357 (4)	0.35975 (4)	0.14820 (3)	0.05024 (16)	
Sn2	0.52091 (4)	0.59836 (4)	0.10841 (3)	0.04935 (16)	
N1	0.9801 (6)	0.0426 (5)	−0.2942 (4)	0.0634 (15)	
O1	0.6229 (5)	0.2161 (4)	0.0182 (3)	0.0638 (13)	
O2	0.7256 (6)	0.1199 (5)	0.1181 (4)	0.0823 (16)	
O3	1.0365 (6)	0.1364 (5)	−0.4230 (5)	0.0909 (18)	
H3	0.9947	0.1233	−0.3857	0.136*	
O4	0.5571 (4)	0.4355 (4)	0.0470 (3)	0.0547 (11)	
O5	0.6272 (5)	0.5360 (4)	0.2274 (3)	0.0595 (12)	
C1	0.6645 (7)	0.1245 (6)	0.0363 (6)	0.0583 (18)	
C2	0.6344 (7)	0.0194 (6)	−0.0508 (5)	0.0623 (18)	
H2A	0.6435	−0.0478	−0.0268	0.075*	
H2B	0.5444	0.0154	−0.0888	0.075*	
C3	0.7237 (7)	0.0198 (6)	−0.1167 (5)	0.0560 (17)	
C4	0.6932 (8)	0.0674 (7)	−0.1940 (6)	0.070 (2)	
H4	0.6136	0.0975	−0.2079	0.084*	
C5	0.7779 (8)	0.0717 (7)	−0.2520 (6)	0.068 (2)	
H5	0.7540	0.1037	−0.3045	0.082*	
C6	0.8976 (7)	0.0290 (6)	−0.2330 (5)	0.0591 (18)	
C7	0.9282 (8)	−0.0209 (7)	−0.1563 (6)	0.070 (2)	
H7	1.0071	−0.0523	−0.1432	0.084*	
C8	0.8430 (8)	−0.0247 (7)	−0.0990 (6)	0.067 (2)	
H8	0.8661	−0.0579	−0.0471	0.081*	
C9	1.0880 (8)	0.0011 (6)	−0.2867 (5)	0.0654 (19)	
H9	1.1127	−0.0415	−0.2418	0.079*	
C10	1.1754 (7)	0.0177 (6)	−0.3466 (5)	0.0634 (19)	
C11	1.1448 (8)	0.0835 (7)	−0.4122 (6)	0.069 (2)	
C12	1.2303 (9)	0.0984 (8)	−0.4689 (7)	0.084 (2)	
H12	1.2097	0.1415	−0.5138	0.101*	
C13	1.3438 (10)	0.0486 (9)	−0.4572 (7)	0.092 (3)	
H13	1.4007	0.0586	−0.4946	0.110*	
C14	1.3772 (10)	−0.0163 (9)	−0.3913 (7)	0.097 (3)	
H14	1.4558	−0.0489	−0.3842	0.117*	
C15	1.2924 (9)	−0.0321 (8)	−0.3362 (7)	0.086 (3)	
H15	1.3134	−0.0761	−0.2921	0.103*	
C16	0.8584 (7)	0.3962 (8)	0.1767 (6)	0.077 (2)	
H16A	0.8793	0.3752	0.1135	0.092*	
H16B	0.8781	0.4782	0.2025	0.092*	
C17	0.9505 (10)	0.3444 (10)	0.2443 (9)	0.111 (3)	
H17A	1.0393	0.3717	0.2486	0.133*	0.70 (8)
H17B	0.9397	0.2624	0.2161	0.133*	0.70 (8)
H17C	1.0235	0.4005	0.2841	0.133*	0.30 (8)
H17D	0.9854	0.2816	0.2056	0.133*	0.30 (8)
C18	0.932 (5)	0.370 (5)	0.347 (4)	0.108 (10)	0.70 (8)
H18A	0.8425	0.3452	0.3424	0.130*	0.70 (8)
H18B	0.9462	0.4521	0.3760	0.130*	0.70 (8)
C19	1.026 (5)	0.312 (4)	0.413 (4)	0.136 (11)	0.70 (8)
H19A	1.0285	0.2344	0.3777	0.203*	0.70 (8)
H19B	0.9956	0.3129	0.4707	0.203*	0.70 (8)
H19C	1.1120	0.3518	0.4337	0.203*	0.70 (8)
C20	0.512 (3)	0.289 (3)	0.2016 (19)	0.072 (7)	0.66 (3)
H20A	0.4270	0.3017	0.1650	0.087*	0.66 (3)
H20B	0.5168	0.2076	0.1863	0.087*	0.66 (3)
C21	0.5211 (16)	0.3327 (13)	0.3105 (12)	0.086 (6)	0.66 (3)
H21A	0.5249	0.4151	0.3279	0.103*	0.66 (3)
H21B	0.6021	0.3130	0.3477	0.103*	0.66 (3)
C22	0.408 (3)	0.286 (3)	0.3417 (19)	0.102 (8)	0.66 (3)
H22A	0.3282	0.3156	0.3129	0.122*	0.66 (3)
H22B	0.3954	0.2038	0.3163	0.122*	0.66 (3)
C23	0.431 (4)	0.318 (3)	0.451 (2)	0.145 (11)	0.66 (3)
H23A	0.5072	0.2855	0.4799	0.217*	0.66 (3)
H23B	0.3565	0.2892	0.4678	0.217*	0.66 (3)
H23C	0.4444	0.3993	0.4769	0.217*	0.66 (3)
C18'	0.914 (11)	0.317 (9)	0.329 (8)	0.10 (2)	0.30 (8)
H18C	0.9037	0.2354	0.3180	0.116*	0.30 (8)
H18D	0.8304	0.3453	0.3337	0.116*	0.30 (8)
C19'	1.018 (10)	0.371 (11)	0.427 (8)	0.13 (3)	0.30 (8)
H19D	0.9762	0.4141	0.4746	0.200*	0.30 (8)
H19E	1.0811	0.4212	0.4163	0.200*	0.30 (8)
H19F	1.0601	0.3124	0.4514	0.200*	0.30 (8)
C20'	0.552 (6)	0.286 (7)	0.238 (4)	0.075 (13)	0.34 (3)
H20C	0.5450	0.2035	0.2138	0.090*	0.34 (3)
H20D	0.6054	0.3074	0.3055	0.090*	0.34 (3)
C21'	0.416 (3)	0.322 (3)	0.236 (2)	0.096 (12)	0.34 (3)
H21C	0.3623	0.2996	0.1688	0.115*	0.34 (3)
H21D	0.4228	0.4046	0.2597	0.115*	0.34 (3)
C22'	0.347 (5)	0.272 (6)	0.300 (4)	0.103 (14)	0.34 (3)
H22C	0.2535	0.2657	0.2723	0.123*	0.34 (3)
H22D	0.3719	0.1954	0.2976	0.123*	0.34 (3)
C23'	0.382 (7)	0.343 (7)	0.406 (5)	0.14 (2)	0.34 (3)
H23D	0.4725	0.3400	0.4368	0.217*	0.34 (3)
H23E	0.3287	0.3134	0.4412	0.217*	0.34 (3)
H23F	0.3662	0.4204	0.4088	0.217*	0.34 (3)
C24	0.3385 (7)	0.6054 (7)	0.1451 (6)	0.069 (2)	
H24A	0.2905	0.6593	0.1155	0.083*	
H24B	0.2888	0.5311	0.1146	0.083*	
C25	0.3425 (8)	0.6382 (8)	0.2542 (6)	0.077 (2)	
H25A	0.3828	0.7159	0.2844	0.093*	
H25B	0.3965	0.5891	0.2862	0.093*	
C26	0.2068 (9)	0.6289 (9)	0.2717 (8)	0.102 (3)	
H26A	0.1530	0.6781	0.2396	0.122*	
H26B	0.1665	0.5513	0.2413	0.122*	
C27	0.2089 (13)	0.6614 (12)	0.3807 (9)	0.144 (5)	
H27A	0.2504	0.6060	0.4109	0.216*	
H27B	0.1209	0.6634	0.3868	0.216*	
H27C	0.2567	0.7351	0.4130	0.216*	
C28	0.6575 (8)	0.7411 (7)	0.1316 (6)	0.078 (2)	
H28A	0.6073	0.8061	0.1323	0.094*	0.66 (3)
H28B	0.7134	0.7541	0.1980	0.094*	0.66 (3)
H28C	0.6170	0.7986	0.1023	0.094*	0.34 (3)
H28D	0.6990	0.7753	0.2012	0.094*	0.34 (3)
C29	0.7475 (19)	0.7479 (17)	0.0664 (13)	0.089 (6)	0.66 (3)
H29A	0.6982	0.7264	−0.0025	0.106*	0.66 (3)
H29B	0.7889	0.8250	0.0830	0.106*	0.66 (3)
C30	0.849 (2)	0.6678 (15)	0.0837 (13)	0.090 (6)	0.66 (3)
H30A	0.8063	0.5910	0.0674	0.109*	0.66 (3)
H30B	0.8967	0.6893	0.1529	0.109*	0.66 (3)
C31	0.943 (3)	0.670 (3)	0.021 (2)	0.109 (8)	0.66 (3)
H31A	0.8973	0.6827	−0.0410	0.163*	0.66 (3)
H31B	0.9799	0.5989	0.0082	0.163*	0.66 (3)
H31C	1.0121	0.7308	0.0548	0.163*	0.66 (3)
C29'	0.757 (4)	0.682 (3)	0.077 (3)	0.082 (10)	0.34 (3)
H29C	0.7933	0.6256	0.1093	0.098*	0.34 (3)
H29D	0.7085	0.6412	0.0099	0.098*	0.34 (3)
C30'	0.868 (3)	0.758 (3)	0.071 (2)	0.085 (12)	0.34 (3)
H30C	0.9201	0.7908	0.1390	0.102*	0.34 (3)
H30D	0.8295	0.8203	0.0489	0.102*	0.34 (3)
C31'	0.965 (5)	0.718 (4)	0.011 (4)	0.096 (13)	0.34 (3)
H31D	1.0305	0.6814	0.0469	0.143*	0.34 (3)
H31E	1.0050	0.7821	−0.0016	0.143*	0.34 (3)
H31F	0.9190	0.6647	−0.0507	0.143*	0.34 (3)
C32	0.6961 (9)	0.6034 (8)	0.3245 (6)	0.097 (3)	
H32A	0.7842	0.6253	0.3252	0.145*	
H32B	0.6974	0.5596	0.3709	0.145*	
H32C	0.6530	0.6704	0.3425	0.145*	

### Atomic displacement parameters (Å^2^)

**Table:**

	*U*^11^	*U*^22^	*U*^33^	*U*^12^	*U*^13^	*U*^23^
Sn1	0.0542 (3)	0.0548 (3)	0.0449 (3)	0.0153 (2)	0.0181 (2)	0.0133 (2)
Sn2	0.0521 (3)	0.0467 (3)	0.0438 (3)	0.0123 (2)	0.0129 (2)	0.0019 (2)
N1	0.063 (4)	0.068 (4)	0.062 (4)	0.012 (3)	0.024 (3)	0.015 (3)
O1	0.081 (3)	0.058 (3)	0.054 (3)	0.025 (3)	0.022 (3)	0.010 (2)
O2	0.100 (4)	0.078 (4)	0.072 (4)	0.024 (3)	0.016 (3)	0.029 (3)
O3	0.089 (4)	0.106 (5)	0.102 (4)	0.032 (4)	0.043 (4)	0.052 (4)
O4	0.066 (3)	0.048 (3)	0.046 (2)	0.023 (2)	0.013 (2)	0.005 (2)
O5	0.068 (3)	0.057 (3)	0.045 (2)	0.014 (2)	0.009 (2)	0.005 (2)
C1	0.061 (4)	0.058 (4)	0.066 (5)	0.020 (4)	0.031 (4)	0.021 (4)
C2	0.068 (5)	0.050 (4)	0.075 (5)	0.010 (3)	0.030 (4)	0.016 (4)
C3	0.064 (4)	0.047 (4)	0.060 (4)	0.009 (3)	0.028 (4)	0.010 (3)
C4	0.067 (5)	0.074 (5)	0.074 (5)	0.022 (4)	0.026 (4)	0.020 (4)
C5	0.074 (5)	0.076 (5)	0.064 (5)	0.022 (4)	0.024 (4)	0.028 (4)
C6	0.066 (5)	0.059 (4)	0.058 (4)	0.015 (4)	0.024 (4)	0.018 (3)
C7	0.066 (5)	0.080 (5)	0.075 (5)	0.028 (4)	0.032 (4)	0.025 (4)
C8	0.074 (5)	0.074 (5)	0.067 (5)	0.024 (4)	0.029 (4)	0.029 (4)
C9	0.073 (5)	0.066 (5)	0.062 (5)	0.016 (4)	0.024 (4)	0.018 (4)
C10	0.068 (5)	0.065 (5)	0.059 (4)	0.008 (4)	0.023 (4)	0.014 (4)
C11	0.066 (5)	0.074 (5)	0.067 (5)	0.007 (4)	0.026 (4)	0.014 (4)
C12	0.086 (6)	0.090 (6)	0.082 (6)	0.004 (5)	0.032 (5)	0.025 (5)
C13	0.088 (7)	0.111 (8)	0.089 (6)	0.012 (6)	0.047 (6)	0.026 (6)
C14	0.083 (6)	0.122 (8)	0.095 (7)	0.026 (6)	0.038 (6)	0.028 (6)
C15	0.076 (6)	0.106 (7)	0.083 (6)	0.025 (5)	0.032 (5)	0.029 (5)
C16	0.060 (5)	0.094 (6)	0.082 (6)	0.018 (4)	0.018 (4)	0.034 (5)
C17	0.085 (7)	0.126 (9)	0.122 (9)	0.008 (6)	0.010 (6)	0.049 (8)
C18	0.090 (19)	0.13 (3)	0.10 (2)	0.00 (2)	0.002 (15)	0.04 (2)
C19	0.140 (19)	0.13 (3)	0.12 (2)	0.03 (2)	−0.012 (17)	0.05 (2)
C20	0.082 (19)	0.077 (11)	0.063 (17)	0.006 (13)	0.040 (13)	0.011 (14)
C21	0.094 (12)	0.100 (11)	0.069 (11)	−0.005 (8)	0.042 (9)	0.015 (8)
C22	0.11 (2)	0.111 (16)	0.079 (19)	−0.015 (16)	0.047 (14)	0.008 (16)
C23	0.15 (3)	0.16 (3)	0.11 (2)	−0.009 (19)	0.027 (19)	0.036 (18)
C18'	0.09 (4)	0.11 (5)	0.09 (4)	−0.01 (5)	0.01 (3)	0.04 (5)
C19'	0.12 (4)	0.13 (7)	0.12 (4)	0.01 (5)	0.00 (3)	0.02 (6)
C20'	0.09 (4)	0.08 (2)	0.06 (3)	0.00 (2)	0.03 (2)	0.02 (3)
C21'	0.10 (3)	0.10 (2)	0.08 (2)	−0.001 (18)	0.03 (2)	0.017 (17)
C22'	0.11 (4)	0.11 (3)	0.09 (3)	−0.01 (3)	0.03 (2)	0.02 (3)
C23'	0.15 (6)	0.16 (5)	0.11 (5)	−0.01 (4)	0.03 (4)	0.04 (4)
C24	0.062 (5)	0.073 (5)	0.070 (5)	0.017 (4)	0.022 (4)	0.010 (4)
C25	0.076 (5)	0.088 (6)	0.085 (6)	0.027 (4)	0.040 (5)	0.030 (5)
C26	0.095 (7)	0.112 (8)	0.115 (8)	0.017 (6)	0.058 (6)	0.031 (6)
C27	0.165 (12)	0.182 (13)	0.139 (11)	0.055 (10)	0.110 (10)	0.065 (10)
C28	0.073 (5)	0.072 (5)	0.084 (6)	−0.001 (4)	0.014 (5)	0.020 (5)
C29	0.092 (14)	0.075 (11)	0.095 (11)	−0.019 (10)	0.004 (9)	0.040 (10)
C30	0.091 (13)	0.073 (11)	0.108 (12)	0.007 (10)	0.023 (11)	0.029 (9)
C31	0.106 (16)	0.098 (19)	0.123 (17)	−0.022 (14)	0.050 (13)	0.014 (15)
C29'	0.08 (2)	0.07 (2)	0.10 (2)	0.00 (2)	0.018 (19)	0.033 (18)
C30'	0.08 (2)	0.08 (2)	0.10 (2)	−0.002 (16)	0.025 (16)	0.031 (16)
C31'	0.09 (3)	0.10 (4)	0.11 (3)	−0.01 (3)	0.05 (2)	0.03 (3)
C32	0.089 (6)	0.096 (7)	0.062 (5)	0.011 (5)	−0.005 (4)	−0.030 (5)

### Geometric parameters (Å, °)

**Table:**

Sn1—O4	2.022 (4)	C21—H21B	0.9700
Sn1—C20	2.10 (4)	C22—C23	1.49 (4)
Sn1—C16	2.117 (8)	C22—H22A	0.9700
Sn1—O1	2.169 (4)	C22—H22B	0.9700
Sn1—C20'	2.21 (7)	C23—H23A	0.9600
Sn1—O5	2.227 (4)	C23—H23B	0.9600
Sn2—O4	2.051 (4)	C23—H23C	0.9600
Sn2—O4^i^	2.127 (4)	C18'—C19'	1.54 (16)
Sn2—C24	2.129 (7)	C18'—H18C	0.9700
Sn2—C28	2.129 (8)	C18'—H18D	0.9700
Sn2—O5	2.148 (5)	C19'—H19D	0.9600
N1—C9	1.260 (9)	C19'—H19E	0.9600
N1—C6	1.426 (9)	C19'—H19F	0.9600
O1—C1	1.281 (8)	C20'—C21'	1.52 (8)
O2—C1	1.222 (8)	C20'—H20C	0.9700
O3—C11	1.333 (9)	C20'—H20D	0.9700
O3—H3	0.8200	C21'—C22'	1.53 (7)
O4—Sn2^i^	2.127 (4)	C21'—H21C	0.9700
O5—C32	1.432 (8)	C21'—H21D	0.9700
C1—C2	1.512 (10)	C22'—C23'	1.51 (7)
C2—C3	1.505 (9)	C22'—H22C	0.9700
C2—H2A	0.9700	C22'—H22D	0.9700
C2—H2B	0.9700	C23'—H23D	0.9600
C3—C4	1.368 (10)	C23'—H23E	0.9600
C3—C8	1.386 (10)	C23'—H23F	0.9600
C4—C5	1.382 (10)	C24—C25	1.520 (10)
C4—H4	0.9300	C24—H24A	0.9700
C5—C6	1.380 (10)	C24—H24B	0.9700
C5—H5	0.9300	C25—C26	1.527 (11)
C6—C7	1.379 (10)	C25—H25A	0.9700
C7—C8	1.379 (10)	C25—H25B	0.9700
C7—H7	0.9300	C26—C27	1.523 (14)
C8—H8	0.9300	C26—H26A	0.9700
C9—C10	1.460 (10)	C26—H26B	0.9700
C9—H9	0.9300	C27—H27A	0.9600
C10—C11	1.385 (11)	C27—H27B	0.9600
C10—C15	1.393 (11)	C27—H27C	0.9600
C11—C12	1.403 (11)	C28—C29	1.514 (19)
C12—C13	1.360 (12)	C28—C29'	1.56 (4)
C12—H12	0.9300	C28—H28A	0.9700
C13—C14	1.383 (13)	C28—H28B	0.9700
C13—H13	0.9300	C28—H28C	0.9700
C14—C15	1.382 (12)	C28—H28D	0.9700
C14—H14	0.9300	C29—C30	1.50 (3)
C15—H15	0.9300	C29—H29A	0.9700
C16—C17	1.485 (12)	C29—H29B	0.9700
C16—H16A	0.9700	C30—C31	1.52 (3)
C16—H16B	0.9700	C30—H30A	0.9700
C17—C18'	1.49 (11)	C30—H30B	0.9700
C17—C18	1.50 (5)	C31—H31A	0.9600
C17—H17A	0.9700	C31—H31B	0.9600
C17—H17B	0.9700	C31—H31C	0.9600
C17—H17C	0.9700	C29'—C30'	1.51 (6)
C17—H17D	0.9700	C29'—H29C	0.9700
C18—C19	1.54 (7)	C29'—H29D	0.9700
C18—H18A	0.9700	C30'—C31'	1.52 (6)
C18—H18B	0.9700	C30'—H30C	0.9700
C19—H19A	0.9600	C30'—H30D	0.9700
C19—H19B	0.9600	C31'—H31D	0.9600
C19—H19C	0.9600	C31'—H31E	0.9600
C20—C21	1.51 (3)	C31'—H31F	0.9600
C20—H20A	0.9700	C32—H32A	0.9600
C20—H20B	0.9700	C32—H32B	0.9600
C21—C22	1.53 (3)	C32—H32C	0.9600
C21—H21A	0.9700		
			
O4—Sn1—C20	107.0 (8)	C22—C21—H21A	108.6
O4—Sn1—C16	110.7 (3)	C20—C21—H21B	108.6
C20—Sn1—C16	141.6 (7)	C22—C21—H21B	108.6
O4—Sn1—O1	81.82 (17)	H21A—C21—H21B	107.6
C20—Sn1—O1	93.4 (10)	C23—C22—C21	112 (2)
C16—Sn1—O1	98.6 (3)	C23—C22—H22A	109.2
O4—Sn1—C20'	121.8 (15)	C21—C22—H22A	109.2
C20—Sn1—C20'	15.7 (15)	C23—C22—H22B	109.2
C16—Sn1—C20'	126.1 (14)	C21—C22—H22B	109.2
O1—Sn1—C20'	100.2 (19)	H22A—C22—H22B	107.9
O4—Sn1—O5	71.85 (16)	C17—C18'—C19'	112 (8)
C20—Sn1—O5	91.6 (11)	C17—C18'—H18C	109.3
C16—Sn1—O5	93.4 (3)	C19'—C18'—H18C	109.3
O1—Sn1—O5	153.52 (18)	C17—C18'—H18D	109.3
C20'—Sn1—O5	92 (2)	C19'—C18'—H18D	109.3
O4—Sn2—O4^i^	73.39 (19)	H18C—C18'—H18D	108.0
O4—Sn2—C24	113.0 (3)	C18'—C19'—H19D	109.5
O4^i^—Sn2—C24	96.7 (2)	C18'—C19'—H19E	109.5
O4—Sn2—C28	121.4 (3)	H19D—C19'—H19E	109.5
O4^i^—Sn2—C28	98.7 (3)	C18'—C19'—H19F	109.5
C24—Sn2—C28	125.6 (3)	H19D—C19'—H19F	109.5
O4—Sn2—O5	73.00 (17)	H19E—C19'—H19F	109.5
O4^i^—Sn2—O5	146.34 (17)	C21'—C20'—Sn1	115 (4)
C24—Sn2—O5	98.0 (3)	C21'—C20'—H20C	108.6
C28—Sn2—O5	97.1 (3)	Sn1—C20'—H20C	108.6
C9—N1—C6	121.4 (7)	C21'—C20'—H20D	108.6
C1—O1—Sn1	114.1 (4)	Sn1—C20'—H20D	108.6
C11—O3—H3	109.5	H20C—C20'—H20D	107.6
Sn1—O4—Sn2	113.17 (19)	C20'—C21'—C22'	114 (4)
Sn1—O4—Sn2^i^	140.2 (2)	C20'—C21'—H21C	108.7
Sn2—O4—Sn2^i^	106.61 (19)	C22'—C21'—H21C	108.7
C32—O5—Sn2	126.5 (5)	C20'—C21'—H21D	108.7
C32—O5—Sn1	128.9 (5)	C22'—C21'—H21D	108.7
Sn2—O5—Sn1	101.95 (17)	H21C—C21'—H21D	107.6
O2—C1—O1	123.5 (7)	C23'—C22'—C21'	112 (5)
O2—C1—C2	120.5 (7)	C23'—C22'—H22C	109.1
O1—C1—C2	116.0 (7)	C21'—C22'—H22C	109.1
C3—C2—C1	111.9 (6)	C23'—C22'—H22D	109.1
C3—C2—H2A	109.2	C21'—C22'—H22D	109.1
C1—C2—H2A	109.2	H22C—C22'—H22D	107.8
C3—C2—H2B	109.2	C22'—C23'—H23D	109.5
C1—C2—H2B	109.2	C22'—C23'—H23E	109.5
H2A—C2—H2B	107.9	H23D—C23'—H23E	109.5
C4—C3—C8	117.3 (6)	C22'—C23'—H23F	109.5
C4—C3—C2	121.7 (7)	H23D—C23'—H23F	109.5
C8—C3—C2	120.9 (7)	H23E—C23'—H23F	109.5
C3—C4—C5	121.6 (7)	C25—C24—Sn2	117.3 (5)
C3—C4—H4	119.2	C25—C24—H24A	108.0
C5—C4—H4	119.2	Sn2—C24—H24A	108.0
C6—C5—C4	120.9 (7)	C25—C24—H24B	108.0
C6—C5—H5	119.6	Sn2—C24—H24B	108.0
C4—C5—H5	119.6	H24A—C24—H24B	107.2
C7—C6—C5	118.0 (7)	C24—C25—C26	112.7 (8)
C7—C6—N1	125.7 (7)	C24—C25—H25A	109.1
C5—C6—N1	116.4 (6)	C26—C25—H25A	109.1
C6—C7—C8	120.6 (7)	C24—C25—H25B	109.1
C6—C7—H7	119.7	C26—C25—H25B	109.1
C8—C7—H7	119.7	H25A—C25—H25B	107.8
C7—C8—C3	121.6 (7)	C27—C26—C25	113.5 (9)
C7—C8—H8	119.2	C27—C26—H26A	108.9
C3—C8—H8	119.2	C25—C26—H26A	108.9
N1—C9—C10	121.9 (7)	C27—C26—H26B	108.9
N1—C9—H9	119.0	C25—C26—H26B	108.9
C10—C9—H9	119.0	H26A—C26—H26B	107.7
C11—C10—C15	119.6 (7)	C26—C27—H27A	109.5
C11—C10—C9	121.2 (7)	C26—C27—H27B	109.5
C15—C10—C9	119.2 (7)	H27A—C27—H27B	109.5
O3—C11—C10	122.4 (7)	C26—C27—H27C	109.5
O3—C11—C12	117.5 (8)	H27A—C27—H27C	109.5
C10—C11—C12	120.1 (8)	H27B—C27—H27C	109.5
C13—C12—C11	118.9 (9)	C29—C28—C29'	32.6 (11)
C13—C12—H12	120.6	C29—C28—Sn2	124.4 (9)
C11—C12—H12	120.6	C29'—C28—Sn2	100.1 (15)
C12—C13—C14	122.0 (9)	C29—C28—H28A	106.2
C12—C13—H13	119.0	C29'—C28—H28A	138.2
C14—C13—H13	119.0	Sn2—C28—H28A	106.2
C15—C14—C13	119.1 (9)	C29—C28—H28B	106.2
C15—C14—H14	120.4	C29'—C28—H28B	96.4
C13—C14—H14	120.4	Sn2—C28—H28B	106.2
C14—C15—C10	120.2 (9)	H28A—C28—H28B	106.4
C14—C15—H15	119.9	C29—C28—H28C	81.9
C10—C15—H15	119.9	C29'—C28—H28C	112.1
C17—C16—Sn1	120.8 (6)	Sn2—C28—H28C	111.8
C17—C16—H16A	107.1	H28A—C28—H28C	27.4
Sn1—C16—H16A	107.1	H28B—C28—H28C	126.3
C17—C16—H16B	107.1	C29—C28—H28D	112.8
Sn1—C16—H16B	107.1	C29'—C28—H28D	111.2
H16A—C16—H16B	106.8	Sn2—C28—H28D	112.0
C16—C17—C18'	120 (5)	H28A—C28—H28D	88.3
C16—C17—C18	114 (2)	H28B—C28—H28D	18.1
C18'—C17—C18	24 (3)	H28C—C28—H28D	109.4
C16—C17—H17A	108.8	C30—C29—C28	107.6 (16)
C18'—C17—H17A	121.9	C30—C29—H29A	110.2
C18—C17—H17A	108.8	C28—C29—H29A	110.2
C16—C17—H17B	108.8	C30—C29—H29B	110.2
C18'—C17—H17B	84.6	C28—C29—H29B	110.2
C18—C17—H17B	108.8	H29A—C29—H29B	108.5
H17A—C17—H17B	107.7	C29—C30—C31	110.5 (18)
C16—C17—H17C	109.4	C29—C30—H30A	109.5
C18'—C17—H17C	95.7	C31—C30—H30A	109.5
C18—C17—H17C	75.9	C29—C30—H30B	109.5
H17A—C17—H17C	36.6	C31—C30—H30B	109.5
H17B—C17—H17C	135.0	H30A—C30—H30B	108.1
C16—C17—H17D	109.4	C30'—C29'—C28	117 (3)
C18'—C17—H17D	112.8	C30'—C29'—H29C	108.1
C18—C17—H17D	132.5	C28—C29'—H29C	108.1
H17A—C17—H17D	73.7	C30'—C29'—H29D	108.1
H17B—C17—H17D	36.0	C28—C29'—H29D	108.1
H17C—C17—H17D	107.6	H29C—C29'—H29D	107.3
C17—C18—C19	112 (4)	C29'—C30'—C31'	124 (3)
C17—C18—H17C	36.7	C29'—C30'—H30C	106.3
C19—C18—H17C	100.4	C31'—C30'—H30C	106.3
C17—C18—H18A	109.1	C29'—C30'—H30D	106.3
C19—C18—H18A	109.1	C31'—C30'—H30D	106.3
H17C—C18—H18A	143.1	H30C—C30'—H30D	106.4
C17—C18—H18B	109.1	C30'—C31'—H31D	109.5
C19—C18—H18B	109.1	C30'—C31'—H31E	109.5
H17C—C18—H18B	81.9	H31D—C31'—H31E	109.5
H18A—C18—H18B	107.8	C30'—C31'—H31F	109.5
C21—C20—Sn1	118 (2)	H31D—C31'—H31F	109.5
C21—C20—H20A	107.9	H31E—C31'—H31F	109.5
Sn1—C20—H20A	107.9	O5—C32—H32A	109.5
C21—C20—H20B	107.9	O5—C32—H32B	109.5
Sn1—C20—H20B	107.9	H32A—C32—H32B	109.5
H20A—C20—H20B	107.2	O5—C32—H32C	109.5
C20—C21—C22	114.6 (19)	H32A—C32—H32C	109.5
C20—C21—H21A	108.6	H32B—C32—H32C	109.5

Symmetry codes: (i) −*x*+1, −*y*+1, −*z*.
